# Radioimmunotherapy Combined with Maintenance Anti-CD20 Antibody May Trigger Long-Term Protective T Cell Immunity in Follicular Lymphoma Patients

**DOI:** 10.1155/2013/875343

**Published:** 2013-11-26

**Authors:** Franz Buchegger, Steven M. Larson, Jean-Pierre Mach, Yves Chalandon, Pierre-Yves Dietrich, Anne Cairoli, John O. Prior, Pedro Romero, Daniel E. Speiser

**Affiliations:** ^1^Department of Nuclear Medicine, Lausanne University Hospital, 1011 Lausanne, Switzerland; ^2^Department of Nuclear Medicine, Geneva University Hospitals, 1211 Geneva, Switzerland; ^3^Department of Radiology, Memorial Sloan Kettering Cancer Center, New York, NY 10065, USA; ^4^Department of Biochemistry, University of Lausanne, 1066 Lausanne, Switzerland; ^5^Department of Hematology, Geneva University Hospitals, 1211 Geneva, Switzerland; ^6^Department of Oncology, Geneva University Hospitals, 1211 Geneva, Switzerland; ^7^Department of Oncology, Lausanne University Hospital, 1011 Lausanne, Switzerland; ^8^Ludwig Center, Department of Oncology, Lausanne University Hospital, 1011 Lausanne, Switzerland

## Abstract

Growing evidence suggests that the patient's immune response may play a major role in the long-term efficacy of antibody therapies of follicular lymphoma (FL). Particular long-lasting recurrence free survivals have been observed after first line, single agent rituximab or after radioimmunotherapy (RIT). Rituximab maintenance, furthermore, has a major efficacy in prolonging recurrence free survival after chemotherapy. On the other hand, RIT as a single step treatment showed a remarkable capacity to induce complete and partial remissions when applied in recurrence and as initial treatment of FL or given for consolidation. These clinical results strongly suggest that RIT combined with rituximab maintenance could stabilize the high percentages of patients with CR and PR induced by RIT. While the precise mechanisms of the long-term efficacy of these 2 treatments are not elucidated, different observations suggest that the patient's T cell immune response could be decisive. With this review, we discuss the potential role of the patient's immune system under rituximab and RIT and argue that the T cell immunity might be particularly promoted when combining the 2 antibody treatments in the early therapy of FL.

## 1. Long-Term Complete Remissions of FL Have Been Reported after Either RIT or Rituximab Treatment

Advanced stage follicular lymphoma (FL) cannot be cured by standard chemotherapy [1]. In the frequently slow evolution of the disease, FL tends to become less responsive to chemotherapy, remissions lasting shorter time or the disease transforms to higher grade lymphoma. In recent years, it has been shown repeatedly that allogeneic hematopoietic stem cells transplantation (HSCT) can induce a plateau of tumor free survival and has a curative potential [2–4], suggesting that donor T cells bear the potential to cure FL patients [5]. 

The first, highly efficient anti-CD20 radioimmunotherapy (RIT) has been published in 1993 [6, 7] and single agent rituximab (Mabthera, Rituxan, Roche Ltd, Genentech) treatments were published a few years later [8–11]. Long-term complete remissions (CR) lasting 8 years or more have since been reported after single agent rituximab as well as after RIT [9, 10, 12–15]. These sustained remissions might be a first indication of the curative potential of the 2 antibody based treatments.

There is increasing evidence that the patient's T cells could be particularly involved in these long-term responses, as recently postulated in a letter to the editor [16]. The combination of the 2 treatments, as discussed here, with the particular aim to preserve, stimulate, and study the patient's T cell response would appear attractive notably when being initiated as first line or early treatment of FL.

## 2. The Patient's Immune Response May Be Relevant to Long-Term Tumor Control of FL

The tumor microenvironment shows remarkable adaptations in FL [17]. Tumor cells interact with stromal cells allowing the promotion of tumor growth [18–20]. Cytotoxic T lymphocytes were shown to have a tumor controlling potential, but, once they are present in the tumor microenvironment, they may be inhibited by regulatory T and/or B cells [21–25] or exhausted as shown in melanoma [26] and lymphoma [27].

Conversely, immune response signatures have identified tumor infiltrating T cells, monocytes, and dendritic cells as being predictive of survival of FL [28]. RT-PCR based gene expression profiling results were in agreement with these observations [29]. Interestingly, high numbers of tumor infiltrating FOXP3 positive regulatory T cells were also predictive of improved overall survival [30]. The nature as well as the potential heterogeneity of regulatory T cells remains, however, controversial [31, 32]. Recently, follicular Th cells in the tumor microenvironment have also been implicated in promoting immunosuppression [33]. Thus, contradicting roles have been observed for tumor infiltrating stromal cells [32].

Under rituximab treatment elimination of regulatory B cells (B_reg_, B10) could be an explanation for the observation of favorable T cell responses [24, 34]. Different groups have also studied at the preclinical and clinical level the efficacy of blocking T-cell inhibitory receptors [35]. Anti-CTLA-4 [25, 36] and anti-PD-1 [37, 38] treatments showed interesting results in animals and lymphoma patients. Possibly, this approach may become increasingly successful, similar to the progress in patients with melanoma, lung, and kidney cancers [39–42].

Allogeneic HSCT provides the patient with the immunological graft antilymphoma effect that is not necessarily linked with a graft versus host reactivity [43]. It leads to a plateau in tumor free survival and provides a chance of cure in advanced stage FL [2–4]. In nonmyeloablative allogeneic HSCT, the potential curative response of chemorefractory patients who had a conditioning regimen including ^90^Y-ibritumomab, fludarabine, and cyclophosphamide was interpreted as likely being related to the graft versus lymphoma effect, facilitated by an improved initial disease control, provided by radioimmunotherapy (RIT) [2]. 

Weiner et al. highlighted 3 potential mechanisms for induction of a tumor-antigen-specific immune response [44], namely, antibody dependent cellular cytotoxicity (ADCC), antibody-targeted cross-presentation of tumor antigens [45], and triggering of the anti-idiotypic antibodies [44]. Regarding the latter mechanism, the anti-idiotype antibody response of FL patients after idiotype vaccination was shown to correlate with better overall survival [46]. Such a “vaccinal effect” resulting in the induction of a specific anti-idiotype T cell response has also been observed after rituximab treatment [47]. 

Different preclinical studies further highlighted the potential role of rituximab alone or in combination with vaccines in immune stimulation leading to long-term protection in lymphoma models [48–50]. Stimulation of CD8 T cells has been shown to provide long-lasting antitumor immune protection in syngeneic mouse lymphoma [51–53]. Toll-like receptor stimulation in combination with rituximab treatment has been explored in first clinical studies [54, 55].

## 3. Single Agent Rituximab Treatment Is Able to Stimulate T Cell Responses

Single agent rituximab treatment has been studied in different trials [8–11]. Rituximab is particularly attractive due to its capacity to treat B lymphoma while totally sparing T cells [9]. Rituximab maintenance has been shown to be highly efficient after the second line R-chemotherapy but is currently also proposed after the first line R-chemotherapy with a significant benefit shown in the PRIMA trial [56]. Long-lasting complete responses (CR) have been observed in FL patients responding (67%) in an early SAKK trial to initial, single agent rituximab. 45% of the responding patients after first line rituximab reached 8 years continued CR when randomized into the rituximab maintenance arm [9], even though rituximab maintenance in this early study consisted of only 4 supplemental antibody injections administered every 2nd month. Long-term responses were also described after rituximab standard treatment alone [10].

Upon rituximab treatment of lymphoma, activation of NK cells occurs [57] and presence of high numbers of CD4 and CD8 T cells has been shown as being favorable [58]. CD4 T helper cells, CD8 T cells, and NK cells remained stable under rituximab treatment while helper and NK cells were significantly associated with response and EFS, respectively [59]. A lymphoma idiotype-specific T cell response was described for FL patients treated by rituximab alone, suggesting that this therapy might also act as a vaccine [47]. Tumor-infiltrating T-helper cells are repressed in the tumor environment as compared with the same population in normal lymph nodes, however, it was shown that they retain their polyfunctional potential to respond to vaccinal stimulation [60]. These observations clearly indicate that rituximab can stimulate the patient's antilymphoma T cell response.

Possibly, another set of T cells, the *γδ* T cells may be involved in FL immunity [61, 62]. Rituximab could potentially play an enhancing role with regard to these cells as well [63, 64]. Furthermore, under combined treatment with rituximab and interferon-*α* [65], the presence of CD4^+^ and CD8^+^ T cells was shown to be favorable while in a different study interferon was able to abrogate the negative effect of IL4 on immune cells [58].

Elimination of regulatory B cells (B_reg_, B10) could be a further explanation to the observation of T cell responses under rituximab treatment [24, 34]. In mice, such Il-10 producing B10 cells inhibited lymphoma clearance during anti-CD20 immunotherapy. Stimulation of effector T cells with a TLR3 agonist was able to overcome the negative regulatory effects of B10 cells [34]. Since B10 cells are at least partially eliminated by the anti-CD20 therapy, the B cell mediated immunesuppression may be abrogated or at least weakened.

## 4. Single Agent or Consolidation RIT Induces Long-Term CR at High Frequency

RIT has been shown to be an effective single agent therapy at conventional, nonmyeloablative dosing as well as in high dose therapy both in patients in relapse [6, 7, 66] and as an initial treatment [12, 67]. RIT consolidation after chemotherapy was shown to convert high numbers of partial remissions (PR) into CR and to provide prolonged progression free survival [68, 69], the latter being also confirmed at the molecular level [14, 70]. Regarding the upfront, single agent RIT with ^131^I-tositumomab [12], a 40% 10-year relapse free survival was reported [13]. While anti-CD20 directed RIT also reduced B cells, T cell responses in these patients appeared to be preserved [12]. We observed similarly long-lasting CR in a small series of relapsed indolent lymphoma patients treated between 1999 and 2001 with ^131^I-tositumomab [71], with 6 out of 12 patients showing continued CR at currently 12 years after RIT [72]. 

The particular high efficacy of RIT is probably not explained by the delivered radiation dose or by the 2 injections of anti-CD20 antibody given at reduced dose. Rather, the combination of both may be important. Indeed, it has been shown that irradiation can contribute to the induction of durable antitumor response in mouse models of lymphoma [52, 53]. It seems thus reasonable to hypothesize that the long-term efficacy of RIT is dependent on the activation and/or the functional preservation of the patient's T cell antilymphoma response [12, 13]. Furthermore, the fact that the two approved anti-CD20 antibodies, tositumomab, and ibritumomab are of murine origin implies that the tumor cells are targeted with murine antigens, which can be recognized in their processed form by the patient's T lymphocytes [73] via cross-presentation by antigen presenting cells, possibly together with antigens from dying tumor cells [45]. CRs induced by RIT might liberate the patient's T cells immunity from the immunosuppressive state imposed by live tumor masses [26] and B_reg_ cells [24]. 

## 5. The Combination of Two Efficient Antibody Based Treatments May Limit Toxicity While the Long-Term Application (Maintenance) of Rituximab Should Favor the Development of an Immune Response 

Major efforts in cancer treatment are currently directed into development of immunotherapy. In this respect, one might consider rituximab treatment as a lucky punch since rituximab treatment is efficient in patients, has moderate side effects, and has been shown to activate NK cells [57]. Rituximab can have a vaccination effect inducing anti-idiotype T cell responses [47], and rituximab treatment alone is able to lead to long-lasting CR in FL patients [9]. While the underlying mechanisms of the efficacy of rituximab are not yet clearly elucidated, it might well be multifunctional beyond T cells responses.

There are different reasons to combine RIT with rituximab maintenance. First, long-term results have been described for both treatments. Both treatments are of moderate toxicity and present a favorable potential of immune response triggering. RIT tumor bulk reduction is known to be highly efficient and could thus potentially reduce tumor mediated immunesuppression. RIT in combination with a long-term treatment, such as rituximab maintenance known to trigger the immune system, could also have a higher chance to build up a robust antitumor immune response and lead to longer recurrence free survival.

We hypothesize that the patient's T cells would mediate the long term efficacy of rituximab and RIT. This hypothesis is only supported by indirect evidence of observations in patients and preclinical studies. Nevertheless, different studies have shown that rituximab can stimulate the patient's T cells and have a vaccination effect while preclinical studies have shown that such effects can be decisive for cure in syngeneic mouse models. In that sense the hypothesis of a major T cell involvement in antibody treatment of FL joins other effects of rituximab that is clearly able to elicit direct apoptosis, ADCC, CDC, or opsonization, although it remains to be determined which of these mechanisms are decisive in patients. Regarding RIT, we argue that the radiation dose given to tumor with nonmyeloablative RIT and the 2 modest amounts of unlabeled antibody are most likely insufficient to explain its long-term efficacy when given as monotherapy upfront [13] or in relapse [69] of FL or indolent lymphoma. These long-term efficacies of RIT and rituximab in indolent lymphoma, a disease that cannot be cured in its advanced form by conventional treatment, prompt the search of different mechanisms of efficacy than the common ones. 

 Induction R-chemotherapy such as R-CVP, R-CHOP, R-MCP, and R-CHVP+I has been studied in large, randomized phase III trials in comparison with chemotherapy alone and has shown the advantage of adding rituximab to chemotherapy leading to improved PFS and frequently also OS [74–77], as recently discussed [78]. Adding 2-year rituximab maintenance to such induction therapy was shown in a large phase III trial to improve PFS [56]. Two recent phase III trials showed at least a similar efficacy for combined R-bendamustin treatment compared with R-CHOP [79, 80]. 

In view of the particular high and reliable efficacy of adding rituximab to different chemotherapies and further using it in maintenance, it is intriguing that maintenance treatment with anti-CD20 antibodies has not yet been evaluated in combination with RIT, possibly because RIT and rituximab are developed with different perspectives. An ongoing phase II study (NCT00770224) piloted by SWOG investigates the combined approach of R-CHOP induction chemotherapy followed by ^131^I-tositumomab consolidation and 4-year rituximab maintenance. First, results of a similar front line study combining R-CHOP followed by ^90^Y-radioimmunotherapy and maintenance rituximab over 2 years showed a sustained immune response with regard to T cell counts and memory immune responses while B cells were repressed [81]. A high number of long-term molecular remissions in FL patients were reported in that study as well [81]. 

Radioimmunotherapy in the upfront setting has shown a remarkable capacity to induce high numbers of long-lasting CR. While R-CHOP combined with rituximab maintenance is currently a well-established upfront treatment, the combination of RIT with rituximab has never been studied. RIT is frequently seen as an alternative therapy to rituximab [82, 83]. This is also the case in a new randomized study that compares rituximab treatment with ^131^I-tositumomab therapy in relapsed FL patients (NCT00078598). However, rituximab treatment is a unique biological treatment with minor toxicity given under R-chemotherapy and in maintenance [84–87]. Clearly, RIT as a single step therapy involves a different therapeutic mechanism than rituximab maintenance. Furthermore, RIT presents the unique capacity of inducing high numbers of CR and of converting PR into CR when used in consolidation. This induction of CR notably may be important for the recovery of an effective T cell response since large tumors are able to inhibit the functioning of T cells [88–91].

Different studies have shown that therapeutic options for FL remain open after RIT, including as well collection and transplantation of HSCT [92]. Furthermore, large studies have shown that MDS incidence is not increased after RIT [93, 94] but might rather be related to combination with preceding chemotherapies, particularly when these include fludarabine [95, 96].

Based on the hypothesis that a preserved or stimulated T cell response mediates long-term CR of FL, we argue that the unique capacity of RIT of inducing high numbers of long-lasting CR by its own, particularly when used upfront [12, 13], would merit to be evaluated in combination with maintenance by rituximab or another anti-CD20 antibody [97–99], possibly double antibody treatment [100, 101] or with adjunction of interferon-*α* [65]. Such combinations may have only moderate toxicity for the patient himself and his T cells and may be most beneficial in previously untreated patients. 

Further insight should be obtained by direct monitoring of the patient's lymphoma specific T cells in RIT studies combined with rituximab maintenance. In parallel, other components of the immune system should also be analyzed, particularly B cells, to determine to which degree they remain repressed over prolonged periods after rituximab treatment, as suggested already from the early single agent rituximab study SAKK 35/98 [9] and others [102]. One could hypothesize that the patient's T cell response would possibly be able to eradicate or at least control FL progenitor cells under such a combination of RIT with rituximab [103], a hypothesis supported by the observation of long-term CR after single agent rituximab and single agent RIT.

## 6. Conclusion

We would expect that the combination of RIT with anti-CD20 antibody maintenance in the early treatment of FL has the potential to be most efficient due to the preservation or/and stimulation of the patient's antitumor T cells response. The addition of anti-PD-1 or anti-CTLA-4 antibodies may further enhance immune responses by reducing inhibitory pathways and regulatory cells. Indeed, consistent and repeated T cell stimulation may be achieved by RIT with its capacity to induce high numbers of CR, combined with rituximab maintenance capable of maintaining such CR. This approach may therefore be well suited as novel and readily feasible perspective towards potential cure of FL.

## Abbreviations

FL: Follicular lymphoma
RIT: Radioimmunotherapy
CR: Complete response
PR: Partial response
HSCT: Hematopoietic stem cells transplantation
RT-PCR: Real-time polymerase chain reaction
ADCC: Antibody dependent cellular cytotoxicity
CDC: Complement dependent cytotoxicity
SAKK: “Schweizerische Arbeitsgemeinschaft für klinische Krebsforschung” (Swiss Society for Clinical Cancer Research)
R-CVP: Rituximab, cyclophosphamide, vincristine, and prednisone
R-CHOP: Rituximab, cyclophosphamide, doxorubicin, vincristine, and prednisone
R-MCP: Rituximab-mitoxantrone, chlorambucil, and prednisolone
R-CHVP+I: Rituximab, cyclophosphamide, doxorubicin, etoposide, and prednisolone plus interferon alfa-2a
EFS: Event free survival
PFS: Progression free survival
OS: Overall survival.
